# Supplementary figures and images for: Data-based stochastic modeling reveals sources of activity bursts in single-cell TGF-β signaling
Source: PLoS Comput Biol. 2022 Jun 27;18(6):e1010266. doi: 10.1371/journal.pcbi.1010266 (PMC9269928; doi:10.1371/journal.pcbi.1010266)

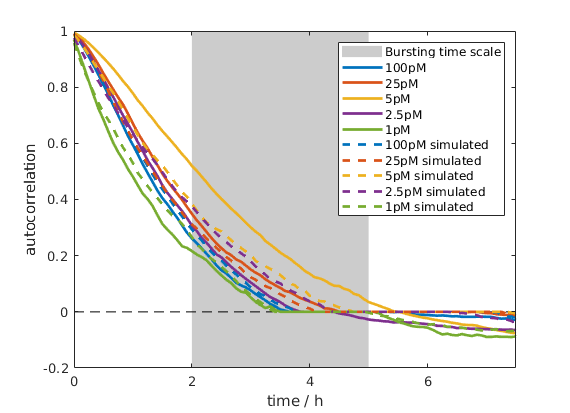

Supplement: S1 Fig — Autocorrelation functions of the nuc/cyt SMAD2 ratio for both the experimental data and the respective simulations (after subtracting the trend) for all 5 considered doses. Autocorrelations for model and data have similar trajectories and reach values close to 0 at similar times fitting within the range considered as intermediate time scale in our definition of bursts (grey shaded area). https://doi.org/10.6084/m9.figshare.19064561. (TIFF) [file pcbi.1010266.s001.tiff]

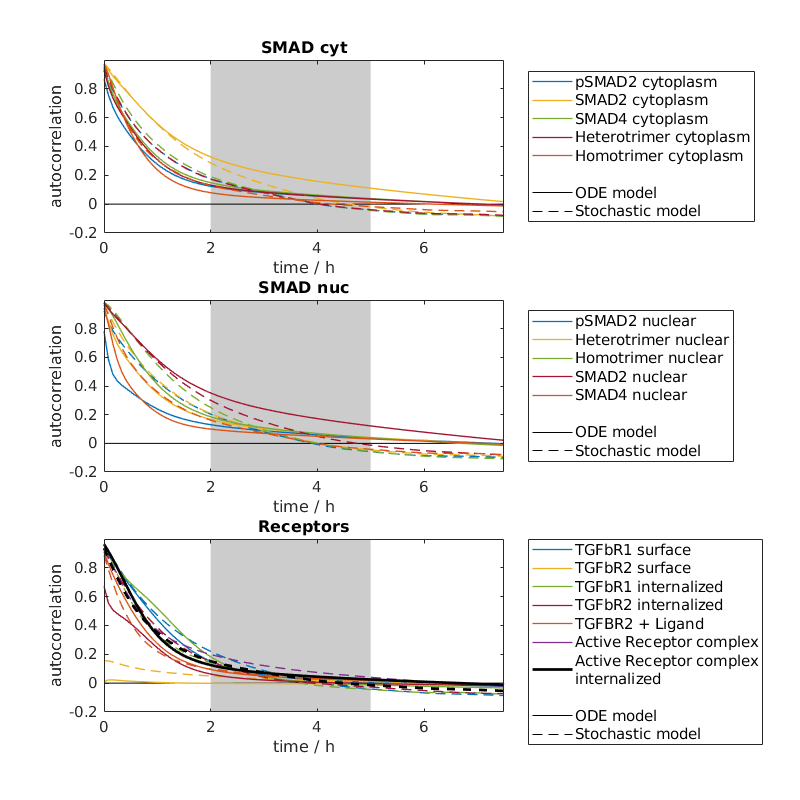

Supplement: S2 Fig — Autocorrelations of individual species within the pathway in the model after subtracting time averages in case of stimulation with 100 pM TGF-β. The autocorrelation trajectories vary among each other, they mostly drop fast in the first 120 minutes but then approach 0 only slowly. In species with slower decay of self-similarity like nuclear and cytoplasmic unphosphorylated SMAD2, the noise led to faster decay. In species with faster decay of self-similarity on the other hand, like nuclear phosphorylated SMAD2 and heterotromer, it led to slower decay. In both cases the stochasticity brings the signal closer to the autocorrelation of the nuc/cyt SMAD2 ratio. No species alone shows an autocorrelation similar to the one of the SMAD2 nuc/cyt ratio (compare S1 Fig). https://doi.org/10.6084/m9.figshare.19064630. (TIFF) [file pcbi.1010266.s002.tiff]

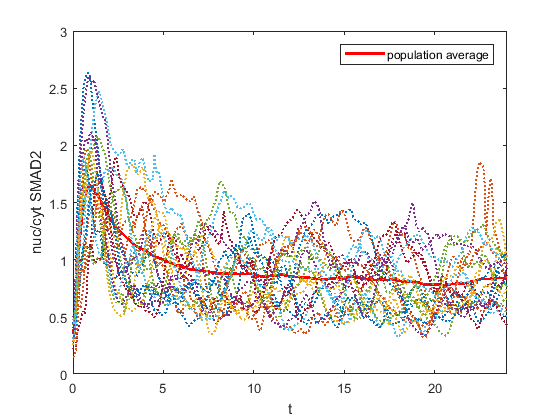

Supplement: S3 Fig — Single cell and population average of nuc/cyt SMAD2 ratio for 100 pM TGF-β stimulation predicted by the OU internalisation model (see S5 Table). In comparison to the CIR internalisation model qualitatively similar bursting is observed in the single cells (compare Fig 3A). https://doi.org/10.6084/m9.figshare.19064663. (TIFF) [file pcbi.1010266.s003.tiff]

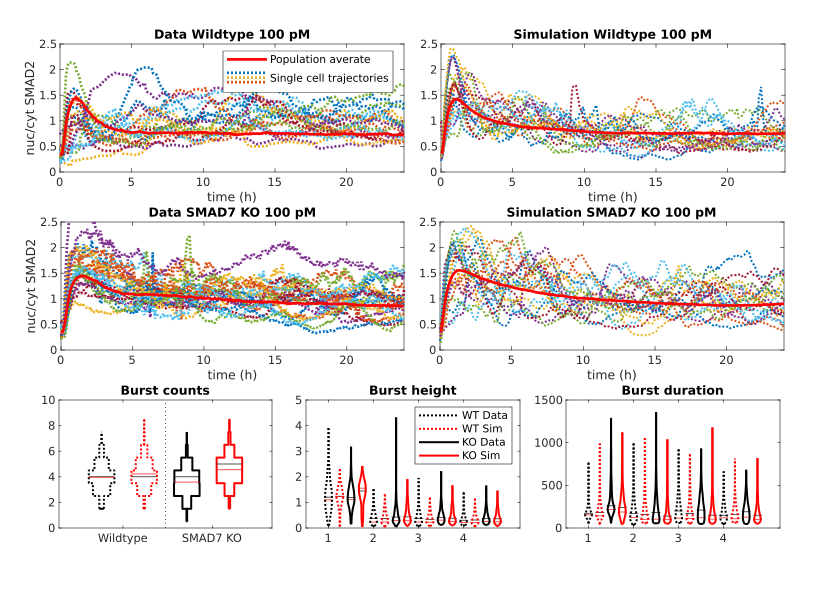

Supplement: S4 Fig — SMAD7 knockout prediction by reducing the parameter of SMAD dependant feedback induction in the internalization model to 30% of its original value in case of a single stimulation with 100 pM TGF-β. Using burst analysis, the model predictions were compared to data from a SMAD7 knockout experiment. Simulation data and burst analysis is presented analog to Fig 5. The population average is well described by the model prediction. Furthermore, the model predicts a slight increase in the mean burst count, amplitude and duration in the SMAD7 knockout. Moreover the burst amplitude and duration variance is predicted to increase. Most of these predictions are indeed observed qualitatively in the SMAD7 knockout compared to wildtype data, although the effect sizes are not very large. The prediction was similarly accurate to those of 5 pM and 25 pM doses of TGF-β (compare Fig 6). https://doi.org/10.6084/m9.figshare.19064687. (TIFF) [file pcbi.1010266.s004.tiff]

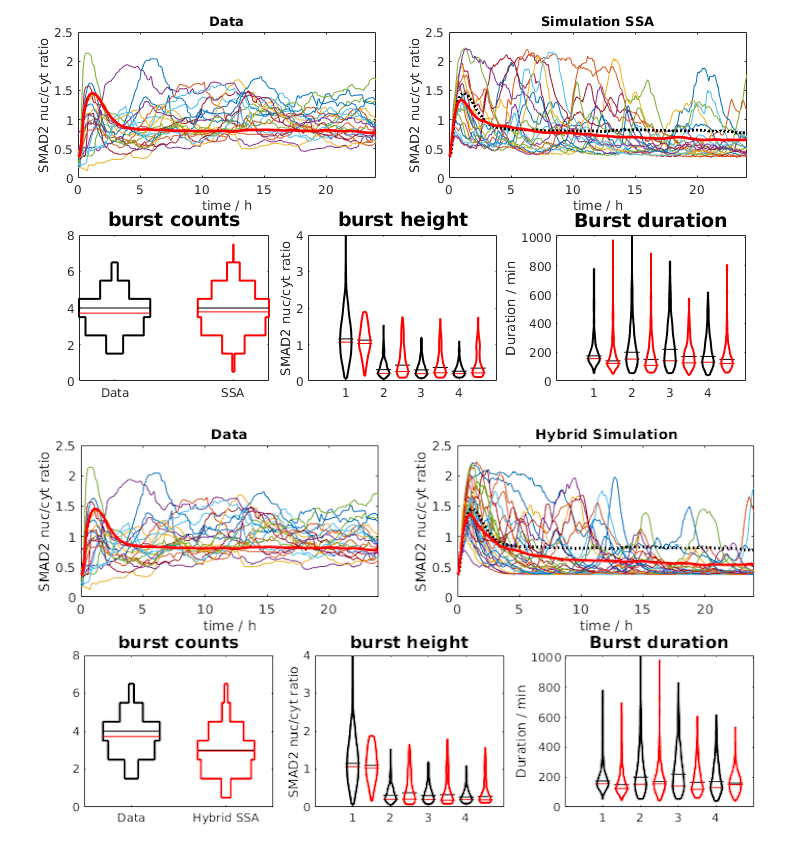

Supplement: S5 Fig — We applied τ-leaping SSA and a hybrid SSA solver to the TGF-β model in case of stimulation with 100 pM TGF-β using the COPASI toolbox [80] and the model from [81]. In a direct comparison between τ-leaping SSA and the internalization CIR model by running 375 paths on the same hardware, the CIR internalization model performed nearly 100x faster (2.2 sec/path vs. 210 sec/path). The hybrid approach combined SSA with a Runge-Kutta method and selected the stochastically treated species based on the number of particles, which also correlates with the expected variance. The publicly available hybrid solver has been much slower than our approach. Simulation data and burst analysis is presented analog to Fig 5. While the computationally expensive SSA reproduced experimental data relatively accurately, the hybrid approach failed to accurately describe the population average and underestimated the number of bursts. Unlike in the CIR internalization model, in both the full SSA and the hybrid method, the nuc/cyt SMAD2 ratio tends to stick to the level before stimulation once the trajectory reached this level again and subsequent stochastic events were less frequently observed. This behaviour was even more prominent in the hybrid model. Moreover the full SSA model predicts a higher number of bursts with high amplitude, in comparison to both data and CIR internalization model, which all attain a similar maximum level. Both SSA models overestimate the heights of later bursts, which was not observed in the CIR internalization model. The predicted bursts thus vary significantly from the ones observed in the experimental data and those predicted by the CIR internalization model. https://doi.org/10.6084/m9.figshare.19064702. (TIFF) [file pcbi.1010266.s005.tiff]

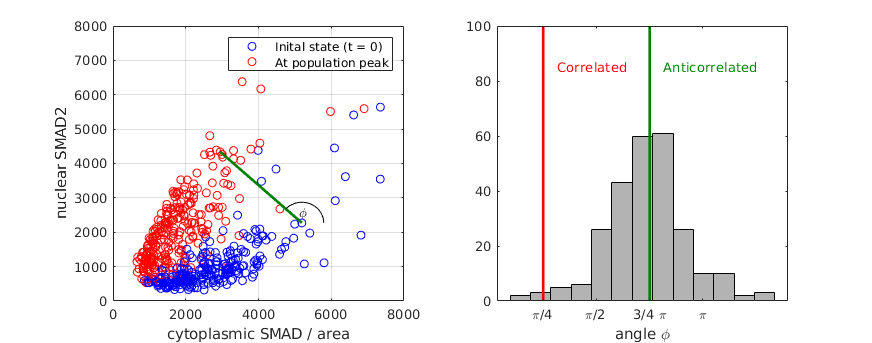

Supplement: S6 Fig — To validate that the bursting events in the nuc/cyt SMAD2 ratio do not arise from technical artefacts, we analyzed the behavior of cytoplasmic as well as nuclear SMAD2 pools in trajectories of single cells. If the bursts are related to nuclear translocation, the two pools must be negatively correlated in single cells during bursting events. Even though over all cells and considered time points, nuclear and cytoplasmic SMAD2 are positively correlated, in the progress of the initial burst, they are indeed negatively correlated. In the scatter plot on the left each trajectory is represented by a blue dot and a red dot encoding nuclear and cytoplasmic SMAD2 at the time before and within a burst event respectively. The histogram on the right shows the distribution of angles of the line connecting the initial state to the burst peak in the cell population, clearly indicating that for most cells, the increase in nuclear SMAD2 matched the decrease of the cytoplasmic SMAD2. This excludes the existence of globally correlated measurement noise and suggests that nuclear and cytoplasmic SMAD2 is negatively correlated within bursts. https://doi.org/10.6084/m9.figshare.19064699. (TIFF) [file pcbi.1010266.s006.tiff]

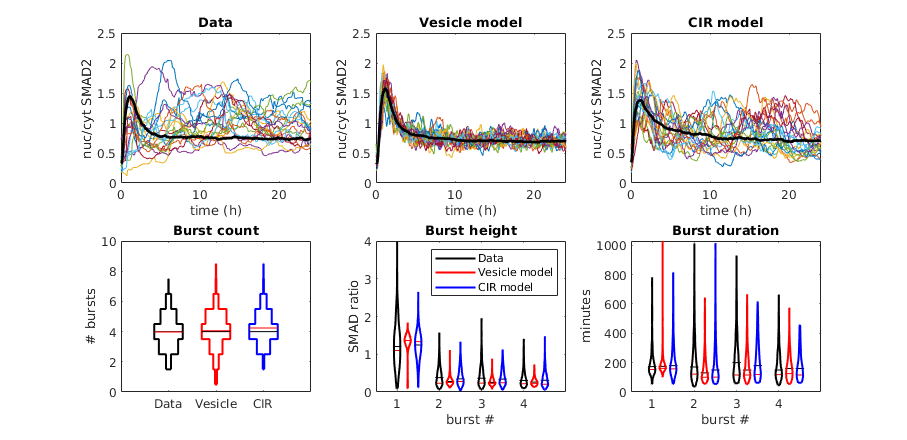

Supplement: S7 Fig — To assess the role of receptor sorting into vesicles we considered an alternative model. This vesicle model assumed the receptors to be attributed to a particular area on the cell surface. We randomly decided whether each vesicle got internalized or not. For each vesicle that gets internalized, all attributed receptors were internalized, independent of their binding states. For simplicity, after internalisation, the receptors are assumed to be released and recycled or degraded according to the ODE model. Thus the underlying hypothesis of vesicles is not yet consistently translated into the new model. Still, even without parameter fitting, the number of bursting events agreed well to the experimental data. Simulation data and burst analysis is presented analog to Fig 5. While the variance predicted by this model is clearly underestimated, the average height and duration of bursting events were well predicted (see also S8 Table). The variance of the burst properties though, were underestimated. While the results of the vesicle model are promising, further research and modeling are required to better describe the data. https://doi.org/10.6084/m9.figshare.19064675.v1. (TIFF) [file pcbi.1010266.s007.tiff]

**Ligand degradation**

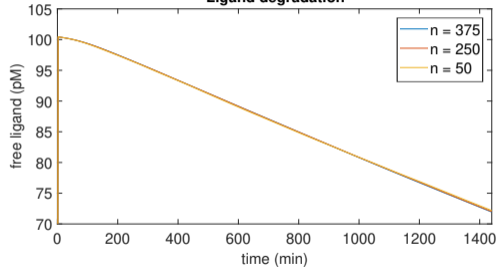

**Differences in ligand degradation**

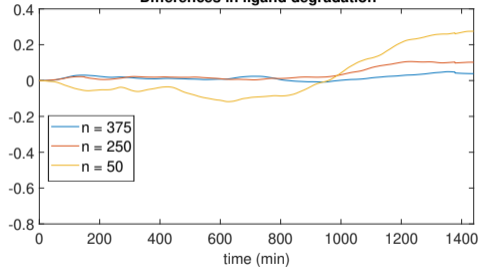

Supplement: S8 Fig — The free ligand concentration in the CIR internalization model in case of stimulation with 100 pM TGF-β for different population sizes (left) and their difference from a reference ligand concentration computed by assuming population size n = 750 (right). Temporal fluctuations in the ligand concentration average out for large population sizes. https://doi.org/10.6084/m9.figshare.19064666. (PDF) [file pcbi.1010266.s008.pdf]
