# Supplementary material for: Data-based stochastic modeling reveals sources of activity bursts in single-cell TGF-β signaling
Source: PLoS Comput Biol. 2022 Jun 27;18(6):e1010266. doi: 10.1371/journal.pcbi.1010266 (PMC9269928; doi:10.1371/journal.pcbi.1010266)
Supplement: S1 Table — Description of the kinetic parameters used in the model and their names in the code. Indications are given when kinetic parameters are used to introduce noise in a block model. The Parameters P25–P29 are used for input scaling and are therefore not considered kinetic parameters. https://doi.org/10.6084/m9.figshare.20012480. (PDF) [file pcbi.1010266.s009.pdf]

	name in the code	description	block model
P_2	K-mran	Smad 7 induction hill parameter (Michaelis constant)	
P_3	R1-total	total number of TGFBR1 receptors	synthesis
P_4	R2-total	total number of TGFBR2 receptors	synthesis
P_5	S2-export-from-nuc	rate of SMAD2 export from the nucleus to the cytoplasm	
P_6	S2-import-to-nuc	rate of SMAD2 import from the cytoplasm to the nucleus	
P_7	S2-total	total number of SMAD2 proteins	
P_8	S4-export-from-nuc	rate of SMAD4 export from the nucleus to the cytoplasm	
P_9	S4-import-to-nuc	rate of SMAD4 import from the cytoplasm to the nucleus	
P_{10}	S4-total	total number of SMAD4 proteins	
P_{11}	Trimer-import-to-nuc	rate of SMAD trimer import to the nucleus	
P_{12}	export-cytoplasm	rate of SMAD7 export from the cell	
P_{13}	hill-fact1	SMAD7 induction hill parameter (hill coefficient)	
P_{14}	index-active-Rec-internalize	internalization rate of activated receptor complexes	internalization
P_{15}	index-induced-R2-deg	decay rate of SMAD7 inactivated TGFBR2 receptors	
P_{16}	index-induced-ligand-deg	decay rate of TGF-β bound by inactivated TGFBR2 receptor	
P_{17}	index-k-out-1-relative-speed	speed of the export of TGFBR1 out of the cell relative to its import into the cell	
P_{18}	index-k-out-2-relative-speed	speed of the export of TGFBR2 out of the cell relative to its import into the cell	
P_{19}	index-kb-R1	speed of the unbinding of TGFBR1 from the ligand relative to its binding from the ligand	
P_{20}	index-kb-R2	speed of the unbinding of TGFBR2 from the ligand relative to its binding from the ligand	
P_{21}	index-kb-homotrimer	rate of the unbinding of SMAD homotrimers into single SMADs	
P_{22}	index-kf-homotrimer	rate of the binding of single SMADs to homotrimers	
P_{23}	index-seq-kb	speed of the unbinding of SMAD7 to activated receptor complexes relative to its unbinding	
P_{24}	index-trimer-dephos	decay rate of pSMAD within a trimer	
P_{25}	k1	dose constant of 1 pM stimulation	
P_{26}	k2	dose constant of 2.5 pM stimulation	
P_{27}	k3	dose constant of 5 pM stimulation	
P_{28}	k4	dose constant of 25 pM stimulation	
P_{29}	k5	dose constant of 100 pM stimulation	
P_{34}	k-Dephos	dephosphorylation rate of SMAD	
P_{35}	k-S7-protein	synthesis rate of SMAD7 from mRNA (translation)	
P_{36}	k-disso-Active-Rec	dissociation rate of active receptor complexes	end. traffic
P_{37}	k-in-1	internalization rate of TGFBR1 (shutteling)	internalization
P_{38}	k-in-2	internalization rate of TGFBR2 (shutteling)	internalization
P_{39}	k-induced-S7-production	rate of the SMAD signaling induced production of SMAD7	
P_{40}	k-medium	cell volume	
P_{41}	k-phosphorylation	phosphorylation rate of SMAD2 to pSMAD2	
P_{42}	kb-trimmer	dissociation rate of SMAD trimers to SMADs	
P_{43}	kdeg-R1	degradation rate of TGFBR1 receptors	degradation
P_{44}	kdeg-R2	degradation rate of TGFBR2 receptors	degradation
P_{45}	kdeg-S2	degradation rate of SMAD2 proteins	
P_{46}	kdeg-S4	degradation rate of SMAD4 proteins	
P_{47}	kdeg-S7	degradation rate of SMAD7 proteins	degradation
P_{48}	kf-R1-activation	rate of TGFBR1 binding to activated TGFBR2 complexes	receptor/ligand
P_{49}	kf-R2-activation	rate of TGFBR2 binding to the ligand	receptor ligand
P_{50}	kf-Seq-S7-Rec	rate of SMAD7 binding to activated receptor complexes	
P_{51}	kf-trimmer	rate of SMAD binding to build SMAD trimers	
P_{52}	kin-deg-Ligand	degradation rate of TGF-β	
P_{53}	kmRNA1deg-S7	degradation rate of SMAD7 mRNA in the nucleus	
P_{54}	kmRNAdeg-S7	degradation rate of SMAD7 mRNA in the cytoplasm	
P_{55}	mRNA-prod	basal production rate of SMAD7 mRNA (transcription)	synthesis
