# Supplementary material for: Data-based stochastic modeling reveals sources of activity bursts in single-cell TGF-β signaling
Source: PLoS Comput Biol. 2022 Jun 27;18(6):e1010266. doi: 10.1371/journal.pcbi.1010266 (PMC9269928; doi:10.1371/journal.pcbi.1010266)
Supplement: S2 Table — Description of the dynamic variables and their corresponding right hand sides used in the model Eqs (1) and (2). For brevity, the dependence of the dynamic concentrations on i (indicating the cell) is omitted in the descriptions of f1, …, f22. The Hill form in the feedback induction was introduced based on the assumption, that DNA-bounded and unbounded SMAD heterotrimers quickly reach an equilibrium, see [7]. Details about the kinetic and experimental parameters used here can be found in S1 and S3 Tables. https://doi.org/10.6084/m9.figshare.20012225. (PDF) [file pcbi.1010266.s010.pdf]

	description		governing time derivative
L	free TGF-β ligand	$f_L =$	$E_1(t) + \sum_{i=1}^{N} E_7^i\, E_9(t) \frac{y_6^i}{N\, P_{40}^i} - \sum_{i=1}^{N} P_{49}^i E_9(t) \frac{L y_2^i}{N\, P_{40}^i}$
y_1	TGF-β receptor I (TGFBR1) on cell surface	$f_1 =$	$E_2 + E_6 y_7 + P_{15} P_{44} y_9 + P_{16} P_{52} y_9 + P_{17} P_{37} y_3 - P_{37} y_1 - P_{48} y_1 y_6$
y_2	TGF-β receptor II (TGFBR2) on cell surface	$f_2 =$	$E_3 + E_7 E_9(t) y_6 + P_{16} P_{52} y_9 + P_{18} P_{38} y_4 - P_{38} y_2 - P_{49} E_9(t) y_2 L$
y_3	endosomal TGFBR1	$f_3 =$	$-P_{17} P_{37} y_3 + P_{36} y_8 + P_{37} y_1 - P_{43} y_3 + P_{44} y_8 + P_{52} y_8$
y_4	endosomal TGFBR2	$f_4 =$	$-P_{18} P_{38} y_4 + P_{36} y_8 + P_{38} y_2 + P_{43} y_8 - P_{44} y_4 + P_{52} y_8$
y_5	endosomal TGF-β	$f_5 =$	$P_{15} P_{44} y_9 + P_{36} y_8 + P_{43} y_8 + P_{44} y_8 - P_{52} y_5$
y_6	complex of TGF-β and TGFBR2 on cell surface	$f_6 =$	$E_6 y_7 - E_7 E_9(t) y_6 - P_{48} y_1 y_6 + P_{49} E_9(t) y_2 L$
y_7	activated complex of TGF-β, TGFBR1 and TGFBR2 on cell surface	$f_7 =$	$-E_6 y_7 + E_8 y_9 - P_{14} P_{37} y_7 + P_{48} y_1 y_6 - P_{50} y_7 y_{17}$
y_8	activated endosomal complex of TGF-β, TGFBR1 and TGFBR2	$f_8 =$	$P_{14} P_{37} y_7 - P_{36} y_8 - P_{43} y_8 - P_{44} y_8 - P_{52} y_8$
y_9	inactivated complex of TGF-β, TGFBR1, TGFBR2 and SMAD7 on cell surface	$f_9 =$	$-E_8 y_9 - P_{15} P_{44} y_9 - P_{16} P_{52} y_9 + P_{50} y_7 y_{17}$
y_{10}	cytoplasmic phosphorylated SMAD2	$f_{10} =$	$\frac{P_5 y_{18}}{2} - P_6 y_{10} + 3P_{21} P_{42} y_{14} - 3P_{22} P_{51} y_{10}^3 + P_{41} y_8 y_{11} + 2P_{42} y_{13} - P_{45} y_{10} + P_{45} y_{13} + 2P_{45} y_{14} + 2P_{46} y_{13} - 2P_{51} y_{10}^2 y_{12}$
y_{11}	cytoplasmic unphosphorylated SMAD2	$f_{11} =$	$E_4 + \frac{P_5 y_{21}}{2} - P_6 y_{11} - P_{41} y_8 y_{11} - P_{45} y_{11}$
y_{12}	cytoplasmic SMAD4	$f_{12} =$	$E_5 + \frac{P_8 y_{22}}{2} - P_9 y_{12} + P_{42} y_{13} + P_{45} y_{13} - P_{46} y_{12} - P_{51} y_{10}^2 y_{12}$
y_{13}	cytoplasmic heterotrimer of two phosphorylated SMAD2 proteins and one SMAD4 protein	$f_{13} =$	$-(P_{11} + P_{42} + P_{45} + P_{46}) y_{13} + P_{51} y_{10}^2 y_{12}$
y_{14}	cytoplasmic homotrimer of phosphorylated SMAD2	$f_{14} =$	$-P_{11} y_{14} - P_{21} P_{42} y_{14} + P_{22} P_{51} y_{10}^3 - P_{45} y_{14}$
y_{15}	cytoplasmic mRNA of SMAD7	$f_{15} =$	$\frac{P_{12} y_{16}}{2} - P_{54} y_{15}$
y_{16}	nuclear mRNA of SMAD7	$f_{16} =$	$P_{39} \frac{y_{19}^{P_{13}}}{P_2^{P_{13}} + y_{19}^{P_{13}}} + P_{55} - P_{12} y_{16} - P_{53} y_{16}$
y_{17}	cytoplasmic SMAD7 (feedback protein)	$f_{17} =$	$E_8 y_9 + P_{15} P_{44} y_9 + P_{16} P_{52} y_9 + P_{35} y_{15} - P_{47} y_{17} - P_{50} y_7 y_{17}$
y_{18}	nuclear phosphorylated SMAD2	$f_{18} =$	$-P_5 y_{18} + 2P_6 y_{10} + 3P_{22} P_{41} y_{20} - 3P_{21} P_{51} y_{18}^3 + P_{24} P_{34} y_{19} - P_{34} y_{18} + 2P_{42} y_{19} - P_{45} y_{18} + P_{45} y_{19} + 2P_{45} y_{20} + 2P_{46} y_{19} - 2P_{51} y_{18}^2 y_{22}$
y_{19}	nuclear heterotrimer of two phosphorylated SMAD2 proteins and one SMAD4 protein	$f_{19} =$	$2P_{11} y_{13} - P_{24} P_{34} y_{19} - P_{42} y_{19} - P_{45} y_{19} - P_{46} y_{19} + P_{51} y_{18}^2 y_{22}$
y_{20}	nuclear homotrimer of phosphorylated SMAD2	$f_{20} =$	$2P_{11} y_{14} - P_{21} P_{42} y_{20} + P_{22} P_{51} y_{18}^3 - P_{45} y_{20}$
y_{21}	nuclear unphosphorylated SMAD2	$f_{21} =$	$-P_5 y_{21} + 2P_6 y_{11} + P_{24} P_{34} y_{19} + P_{34} y_{18} - P_{45} y_{21}$
y_{22}	nuclear SMAD4	$f_{22} =$	$-P_8 y_{22} + 2P_9 y_{12} + P_{24} P_{34} y_{19} + P_{42} y_{19} + P_{45} y_{19} - P_{46} y_{22} - P_{51} y_{18}^2 y_{22}$
