# Supplementary material for: Data-based stochastic modeling reveals sources of activity bursts in single-cell TGF-β signaling
Source: PLoS Comput Biol. 2022 Jun 27;18(6):e1010266. doi: 10.1371/journal.pcbi.1010266 (PMC9269928; doi:10.1371/journal.pcbi.1010266)
Supplement: S3 Table — Parameters of experimental conditions that were used in the model (see S2 Table). The parameter E1 describes the dose dependent bolus induction. The Parameters E2, E3, E4 and E5 describe the production of TGFBR1, TGFBR2, SMAD2 and SMAD4, respectively. Unbinding from complexes is determined by E6 (TGFBR1 from activated complex of TGF-β, TGFBR1 and TGFBR2 (y7)), E7 (TGFBR2 from activated complex of TGF-β and TGFBR2 (y6)) and E8 (SMAD7 from inactivated complex of TGF-β, TGFBR1, TGFBR2 and SMAD7 (y9)). The parameter E9 introduces a short delay in some reactions for numerical stability. https://doi.org/10.6084/m9.figshare.20012702. (PDF) [file pcbi.1010266.s011.pdf]

$$
\begin{aligned}
E_1(t, 1\ \mathrm{pM}) &= P_{25} Q^+(0.1, t)\, Q^-(2.1, t) & E_1(t, 2.5\ \mathrm{pM}) &= P_{26} Q^+(0.1, t)\, Q^-(2.1, t) \\
E_1(t, 5\ \mathrm{pM}) &= P_{27} Q^+(0.1, t)\, Q^-(2.1, t) & E_1(t, 25\ \mathrm{pM}) &= P_{28} Q^+(0.1, t)\, Q^-(2.1, t) \\
E_1(t, 100\ \mathrm{pM}) &= P_{29} Q^+(0.1, t)\, Q^-(2.1, t) & E_1(t, 2{\times}2.5\ \mathrm{pM}) &= P_{26} Q^+(0.1, t)\, Q^-(2.1, t) \quad + \\
& & & \quad P_{26} Q^+(480.1, t)\, Q^-(482.1, t) \\
E_2 &= P_3 \frac{P_{37}\ P_{43}^2}{P_{17} P_{37} P_{43} + P_{43}^2 + P_{37} P_{43}} & E_3 &= P_4 \frac{P_{38}\ P_{44}^2}{P_{18} P_{38} P_{44} + P_{44}^2 + P_{38} P_{44}} \\
E_4 &= P_7 \frac{P_6 P_{45} + P_5 P_{45} + P_{45}^2}{P_5 + P_{45} + 2 P_6} & E_5 &= P_{10} \frac{P_9 P_{46} + P_8 P_{46} + P_{46}^2}{P_8 + P_{46} + 2 P_9} \\
E_6 &= P_{48} P_{19} & E_7 &= P_{49} P_{20} \\
E_8 &= P_{50} P_{23} & E_9(t) &= Q^+(0.1, t) \\
Q^+(\tau, t) &= \frac{1000}{1000 + 100 \exp(\tau - t)} & Q^-(\tau, t) &= \frac{1000}{1000 + 100 \exp(t - \tau))}
\end{aligned}
$$
