# Supplementary material for: Data-based stochastic modeling reveals sources of activity bursts in single-cell TGF-β signaling
Source: PLoS Comput Biol. 2022 Jun 27;18(6):e1010266. doi: 10.1371/journal.pcbi.1010266 (PMC9269928; doi:10.1371/journal.pcbi.1010266)
Supplement: S4 Table — Formulas of the high order corrections used in the numerical scheme. Mj,ni and Nj,ni denote independent identically distributed standard normal random variables. https://doi.org/10.6084/m9.figshare.20012735.v1. (PDF) [file pcbi.1010266.s012.pdf]

$$
\begin{aligned}
G_{k,n}^{i} &= \sum_{j \in S} \sigma_j \sqrt{p_{j,n}^{i}} \frac{\partial f_k(t_n, L_n, \mathbf{Y}_n^k, \mathbf{P}_n^k)}{\partial p_j} \left[\Delta Z_{j,n}^{i} - \frac{1}{2}\Delta t \Delta B_{j,n}^{i}\right] \\
H_{j,n}^{i} &= \frac{\theta_j \sigma_j (p_{j,0}^{i} - p_{j,n}^{i})}{2\sqrt{p_{j,n}^{i}}} \left[\Delta B_{j,n}^{i} \Delta t - \Delta Z_{j,n}^{i}\right] - \theta_j \sigma_j \sqrt{p_{j,n}^{i}} \left[\Delta Z_{j,n}^{i} - \frac{1}{2}\Delta B_{j,k}^{i} \Delta t\right] \\
\Delta Z_{j,n}^{i} &= \frac{1}{2} \sqrt[3]{\Delta t} \left(\mathcal{M}_{j,n}^{i} + \frac{1}{\sqrt{3}} \mathcal{N}_{j,n}^{i}\right)
\end{aligned}
$$
