# Supplementary material for: Data-based stochastic modeling reveals sources of activity bursts in single-cell TGF-β signaling
Source: PLoS Comput Biol. 2022 Jun 27;18(6):e1010266. doi: 10.1371/journal.pcbi.1010266 (PMC9269928; doi:10.1371/journal.pcbi.1010266)
Supplement: S5 Table — Distance measurements of the OU internalisation model in comparison to the CIR internalisation and the deterministic model to experimental data for a stimulation with 100 pM TGF-β (compare Table 1). The best-fit parameters from fitting the CIR internalisation model were used in the OU internalisation model. Parameters could not be a priori estimated for the OU internalisation model due to instabilities caused by negative stochastic parameter values. The components of the objective function in the OU internalization model are consistent and comparable albeit of an increased magnitude in comparison to the results of the CIR internalization model. https://doi.org/10.6084/m9.figshare.19064483. (PDF) [file pcbi.1010266.s013.pdf]

model	model error											
	burst height				burst duration				count	mean	std.	norm
deterministic	0.12	0.20	0.50	0.57	0.10	0.03	0.33	0.44	0.87	2.75	5.73	11.64
CIR internalization	0.01	0.00	0.00	0.00	0.00	0.00	0.00	0.01	0.02	1.19	0.14	1.39
OU internalization	0.21	0.11	0.03	0.13	0.02	0.04	0.10	0.06	0.39	1.52	0.33	2.58
