# Supplementary material for: Data-based stochastic modeling reveals sources of activity bursts in single-cell TGF-β signaling
Source: PLoS Comput Biol. 2022 Jun 27;18(6):e1010266. doi: 10.1371/journal.pcbi.1010266 (PMC9269928; doi:10.1371/journal.pcbi.1010266)
Supplement: S6 Table — In analogy to simultaneous fitting of an error model proposed in [82], the scaling of the residuals of our cost-function was re-normalized by introducing the rescaling factors sk for the components of the objective function rk minimizing ∑k(rk/sk)2 + 2 log(sk). Factors for all components referring to burst height and those referring to burst duration were assumed equal, respectively. This re-scaled cost-function met the requirements on the variance of the residuals to calculate the log-likelihood (LL) and the Akaike information criterion (AIC) of the block models in case of the 100 pM TGF-β stimulation. The AIC ratio was computed with respect to the AIC of the internalization block model and indicates that the internalization model outperforms the other models also taking the degrees of freedom into account. https://doi.org/10.6084/m9.figshare.19064558. (PDF) [file pcbi.1010266.s014.pdf]

model	-2 LL	k	AIC	$\log_{10}$ AIC ratio
degradation	52.66	9	70.66	-5.48
endosomal	92.98	3	98.98	-11.63
internalization	27.43	9	45.43	0.00
receptor-ligand	110.92	6	122.92	-16.83
synthesis	109.61	9	127.61	-17.84
