# Supplementary material for: Data-based stochastic modeling reveals sources of activity bursts in single-cell TGF-β signaling
Source: PLoS Comput Biol. 2022 Jun 27;18(6):e1010266. doi: 10.1371/journal.pcbi.1010266 (PMC9269928; doi:10.1371/journal.pcbi.1010266)
Supplement: S7 Table — Distance measurements of τ-leaping SSA and a hybrid SSA solver (see S5 Fig) in comparison to the CIR internalization and the deterministic model to experimental data for a stimulation with 100 pM TGF-β (compare Table 1). Distance in terms of burst statistics of both τ-leaping SSA and hybrid SSA is increased in comparison to the CIR internalization model. https://doi.org/10.6084/m9.figshare.19064621. (PDF) [file pcbi.1010266.s015.pdf]

model	model error											
	burst height				burst duration				count	mean	std.	norm
deterministic	0.12	0.20	0.50	0.57	0.10	0.03	0.33	0.44	0.87	2.75	5.73	11.64
CIR internalization	0.01	0.00	0.00	0.00	0.00	0.00	0.00	0.01	0.02	1.19	0.14	1.39
full SSA	0.01	0.02	0.01	0.04	0.01	0.01	0.03	0.01	0.01	4.89	0.15	5.20
hybrid SSA	0.01	0.02	0.16	0.23	0.01	0.01	0.17	0.22	0.35	25.70	0.16	27.01
