# Supplementary material for: Data-based stochastic modeling reveals sources of activity bursts in single-cell TGF-β signaling
Source: PLoS Comput Biol. 2022 Jun 27;18(6):e1010266. doi: 10.1371/journal.pcbi.1010266 (PMC9269928; doi:10.1371/journal.pcbi.1010266)
Supplement: S8 Table — Distance measurements of the vesicle model (see S7 Fig) in comparison to the CIR model and the deterministic model relative to experimental data for stimulation with 100 pM TGF-β (compare Table 1). While the number of bursts predicted by the vesicle model matches the experimental data, the fit in standard deviation and population average is increased compared to the CIR model. https://doi.org/10.6084/m9.figshare.19064660. (PDF) [file pcbi.1010266.s016.pdf]

model	model error											
	burst height				burst duration				count	mean	std.	norm
deterministic	0.12	0.20	0.50	0.57	0.10	0.03	0.33	0.44	0.87	2.75	5.73	11.64
CIR internalization	0.01	0.00	0.00	0.00	0.00	0.00	0.00	0.01	0.02	1.19	0.14	1.39
vesicle model	0.04	0.08	0.10	0.04	0.02	0.03	0.04	0.00	0.02	7.53	0.73	8.61
